# Novel Gemini Surfactant for Binding Eu(III)-Polyoxometalate into Hydrogels and Polymer Latexes

**DOI:** 10.3390/gels8120786

**Published:** 2022-11-30

**Authors:** Marin Micutz, Viorel Circu, Monica Ilis, Teodora Staicu

**Affiliations:** 1 Department of Physical Chemistry, University of Bucharest, 4-12 Regina Elisabeta Blvd., 030018 Bucharest, Romania; 2 Department of Inorganic Chemistry, University of Bucharest, 4-12 Regina Elisabeta Blvd., 030018 Bucharest, Romania

**Keywords:** gemini surfactant, poly(acrylic acid) hydrogel, swelling mechanism, poly(methyl methacrylate) latexes, europium-polyoxometalate

## Abstract

The incorporation of rare-earth ions into polymer matrices can lead to useful materials in various fields such as biomarkers, lasers, luminescent devices, optical storage materials, and so on. Methods of incorporation are either extremely simple, such as mixing the polymer and the ion of interest in adequate solvents, or more sophisticated such as synthesizing predesigned monomers that contain the rare-earth ion or binding the ion on an already formed polymer chain. Cationic gemini surfactants represent a class of surfactants that can be used to incorporate metal-oxygen cluster compounds by means of strong electrostatic interactions. In this study, first, a novel cationic gemini surfactant having double bonds on both side chains was designed and prepared. After characterization, the surfactant was used to synthesize hydrogels with different degrees of crosslinking and also as a surfmer in emulsion polymerization of methyl methacrylate. The resulted polymer matrices were able to bind europium-polyoxometalate Na_9_[EuW_10_O_36_]^.^32H_2_O. In case of luminescent lanthanide ions, changing the microenvironment around the metal ion also changes the intensity of some emission peaks as well as other luminescent parameters. Investigation of emission spectra of Eu^3+^ indicates a decrease in the symmetry of the microenvironment, when the polyanions pass from water to latex, to surfactant solution, and to hydrogel.

## 1. Introduction

Trivalent rare-earth ions are the obvious choice when it comes to developing luminescent materials, especially due to their narrow emission bands in the visible domain. Coordinating rare-earth ions with different organic ligands could increase metal emission by one order of magnitude, making such compounds extremely useful in a wide range of medical or technological applications [1,2,3]. The number of organic ligands designed for this purpose is quite impressive, but only some of them are used extensively because of their excellent luminescent properties and ease of preparation. Thus, lanthanide β-diketone (1,3-diketones) complexes are the most popular and the most studied coordination compounds [4,5]. Being highly stable, these coordinated compounds can be incorporated and mixed with different materials transferring their unique optical properties to the final product. The matrix is not only capable of tuning the luminescent properties of the rare-earth metal but can also provide higher chemical stability, processability, and mechanical strength. Encapsulation of rare-earth complexes in glasses obtained via sol-gel method often increases the photostability of organic luminophores [6,7]. Zeolites, mesoporous silicates, organically modified xerogels are also used for obtaining luminescent hybrid materials [8,9,10].

Luminescence performances of lanthanide containing polyoxometalates, especially europium or therbium decatungstate are also outstanding. Typically, these complexes are soluble in water, but they can also be solubilized in organic solvents by replacing the small inorganic cations with large organic counterions or by using surfactant encapsulation [11,12,13,14,15]. Incorporation of different rare-earth ions into polymers is still intensively studied because of the interesting potential applications of such hybrid materials as luminescent devices, optical storage materials, laser systems, biomarkers, etc., [16,17,18,19]. When the lanthanide ions are chemically bonded or coordinated to the polymer chain, either during the polymerization reaction or after the polymer is formed, the luminescent properties are greatly influenced by the type of interactions between the rare-earth ion and polymer, by the distribution of ions along the polymer chain, though not only by the content of the lanthanide ions in the material [20,21]. Simply mixing the polymers and rare-earth complexes in adequate solvents represents an easy path for obtaining luminescent materials [22]. However, because of the weak interactions between polymer and ion complexes or due to the heterogeneity of the ions distribution into the polymer matrix, such materials could not meet the requirements for many practical applications. Concentration quenching of luminescence is also possible.

In recent years gemini surfactants gained increased interest in numerous fields mainly as a result of their lower values of critical micelle concentration, higher solubilization capacity and better physicochemical properties compared to corresponding classic surfactants bearing only one hydrophilic head group [23]. Cationic gemini surfactants can form very strong adsorption layers by electrostatic interactions with natural colloids and surfaces that are mostly negatively charged by nature. Different classes of cationic gemini surfactants were designed and studied [24,25,26] and their applications varied from usual ones as emulsifiers, wetting agents, lubricants to synthesis of mesoporous materials and gene delivery [27,28,29].

In the present work, we proposed the incorporation of europium(III) ions by means of ionic exchange using an Eu(III)-polyoxometalate derivative, Eu-POM, and a novel gemini dicationic surfactant having double bonds that ensure its covalent linking with polymer matrices. The newly synthesized surfactant can act as a crosslinking agent when a water soluble monomer such as acrylic acid is used, resulting hydrogels, or it can stabilize the polymer particles when emulsion polymerization is performed, offering cationic bonding sites on the polymer particles surface. In this way both luminescent hydrogels and latexes are obtained, with Eu-POM ionically bound to the polymer.

## 2. Results and Discussion

### 2.1. Synthesis and Characterization of 1,1′-(1,10-Decanediyl)-Bis [3-(Undec-10-en-1-yl)-Imidazolium] Bromide Salt, BIBr

**Scheme 1:**
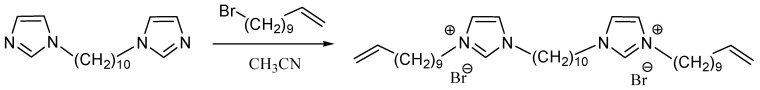
Preparation of bis-imidazolium salt, BIBr.

The dicationic gemini surfactant with chemical structure presented in Scheme 1 contains a decamethylene chain spacer connecting the two imidazolium rings, and two double bonds at the end of the two lateral nonamethylene chains of BIBr.

It is well-known that the surface activity and micellization of gemini surfactants are influenced by their structural features: the two hydrophilic groups, the two or sometimes three hydrophobic groups, and the linkage group between them. The presence of two ionic groups in the surfactant structure can increase its water solubility [30]. For a polymethylene linkage, water solubility and consequently critical micelle concentration (cmc) will increase with the length of the connecting chain due to the fact that a higher distance between the ionic groups ensures a higher degree of ionization [31]. Nevertheless, as the connecting chain length exceeds about 6 methylene groups the cmc decreases [31]. Due to unfavorable interaction between hydrophobic polymethylene chain and aqueous environment it is expected that the hydrophobic chain will accommodate into the micellar core bringing the two ionic groups too close to each other.

**Figure 1:**
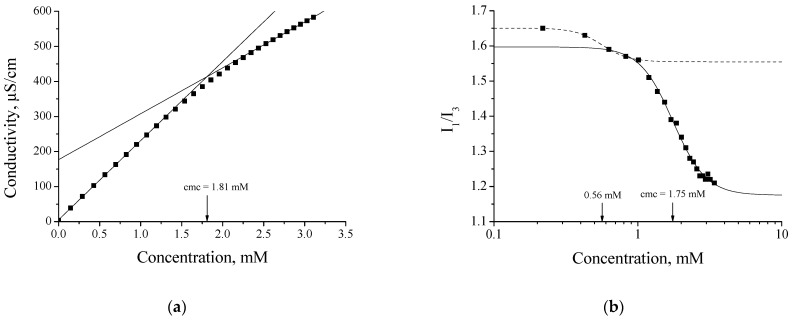
(**a**) Specific conductivity (with linearly fitted dependences before and after cmc) and (**b**) I_1_/I_3_ ratio (with sigmoidal data fitting for the pre-micellar region—dotted line and for determining the real cmc—solid line) vs. concentration of BIBr recorded at room temperature.

For the prepared gemini surfactant, cmc was evaluated by conductometric titration and fluorescence probe method, and similar values were obtained, as can be seen in Figure 1. Thus, conductometric data led to a value for cmc of 1.81 mM, while from fluorescence measurements the same quantity was found to be 1.75 mM. Conductometric data were also processed to evaluate the degree of counterions dissociation in water, *α*, defined as the ratio of the slops above and below cmc on the specific conductivity—surfactant concentration curve. The difference to unity account for the degree of counterions binding, *β*. This last parameter influences the standard Gibbs free energy of micellization, ΔG_m_^0^, for ionic surfactants, according to the following equation [27]:

(1) $$\Delta G_{m}^{0}≅RT(0.5+\beta )\ln X_{cmc}$$

where R is the gas constant, T—temperature, and X_cmc_ is the molar fraction at which the micellization occurs. For cmc measured in mol/L, X_cmc_ is equal to cmc/(cmc + 55.4), where 55.4 represents the moles of water contained in 1 L of pure water at 298.15 K (at which the measurements were carried out). Thus, based on the conductometric titration data and Equation (1), the calculated thermodynamic parameters (*α* = 0.58, *β* = 0.42) allowed obtaining the final value of the standard Gibbs free energy of micellization (−23.6 kJ/mol) which is indicative of the spontaneous character of BIBr micelle formation process. Actually, the same negative value of ΔG_m_^0^ resulted by taking into account cmc value obtained either conductometrically or via fluorescence probe (pyrene) method. In line with these findings, the polar groups of BIBr exhibit a quite large degree of ionization in its micelles, mainly due to the longer polymethylene spacer between such ionogenic moieties.

**Figure 2:**
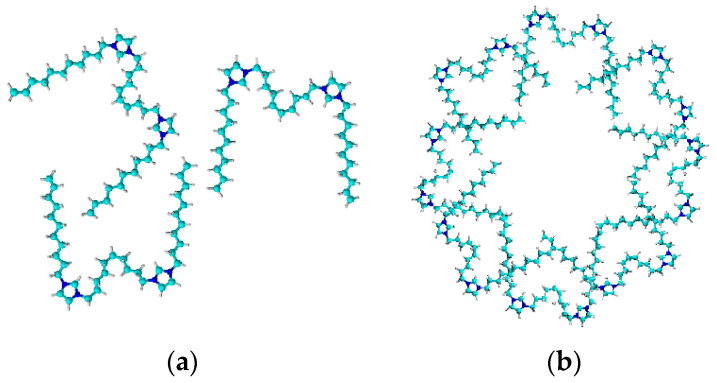
Schematic representation of pre-micellar (**a**) and real micellar (**b**) aggregates of BIBr.

It is worth mentioning that the dependence of I_1_/I_3_ ratio of pyrene on surfactant concentration could be better fitted by means of two sigmoids (Figure 1b). At concentrations below cmc the hydrophobic probe (pyrene molecule) senses a nonpolar microenvironment which can be explained by the formation of small, pre-micellar aggregates (dimmers, trimers, etc.) in the aqueous phase. In fact, these pre-micellar aggregates start to form at concentration around 0.56 mM, while the real micelles begin to exist at much higher concentrations, particularly at 1.75 mM. In Figure 2a schematic representation of micelle formation was depicted. The earlier phenomenon of BIBr self-assembly (small aggregates) during conductometric titration was not detectable conductometrically especially as a result of a negligible number of small counterions (Br^−^) bound to such premicellar aggregates by comparison with that of the total ions in solution mainly responsible for the measurable conductivities.

### 2.2. Hydrogel Characterization

The degree of crosslinking of the obtained hydrogels was assessed by swelling equilibrium measurements. As expected, the larger the amount of crosslinker, the lower the values of equilibrium swelling, SD_eq_, and average molecular mass between two consecutive crosslinkages (M_c_), and consequently the larger the volume fraction of polymer (φ_2_). This is equivalent to say that the degree of crosslinking rises at higher concentrations of BIBr. The parameters of the PAM hydrogels are presented in Table 1. All the poly(acrylic acid) hydrogels synthesized with BIBr are referred to as PAM hydrogels or xerogels (in dry state) in this study. Sample PAM2 was designed by using 2 wt.% (with respect to the amount of acrylic acid, AA) crosslinking agent, and samples PAM4, PAM6, and PAM10 by using 4, 6, and 10 wt.% crosslinker, respectively.

For the highest degree of crosslinking obtained for sample PAM10, the hydrogel hardly swell, and was characterized by a calculated M_c_ value of about 5000 g/mol, which indicates a tightened tridimensional network. On the other hand, for the sample PAM2, with the lowest amount of BIBr, the equilibrium degree of swelling was extremely high, and this hydrogel can be considered a superabsorbent one.

### 2.3. Swelling Behavior

Unlike the equilibrium swelling degree that was evaluated against distilled water, the kinetics of swelling was investigated using an aqueous solution of Eu-POM as swelling solvent. The obtained data were analyzed in accordance with the Korsmeyer–Peppas model [32] which predicts a time dependence of the swelling degree, SD, as follows:

(2) $$SD=k\cdot t^{n}$$

where k is the swelling constant, characterizing the hydrogel network, and *n*—the swelling index, a parameter that gives insights into the swelling mechanism. According to the Korsmeyer–Peppas model, when the value of *n* is approximately 0.5, the swelling follows a Fickian behavior, i.e., the mechanism is controlled by water diffusion into polymer network. For 0.5 < *n* < 1 the swelling becomes non-Fickian or anomalous, while a value above unity corresponds to the Case II diffusion. In contrast with Fickian diffusion, that is specific to swelling of polymers in rubber state (at temperatures well above their glass transition temperature), when the chain flexibility is high, anomalous diffusions appear and the relaxation of polymer network has an important influence upon diffusion.

**Figure 3:**
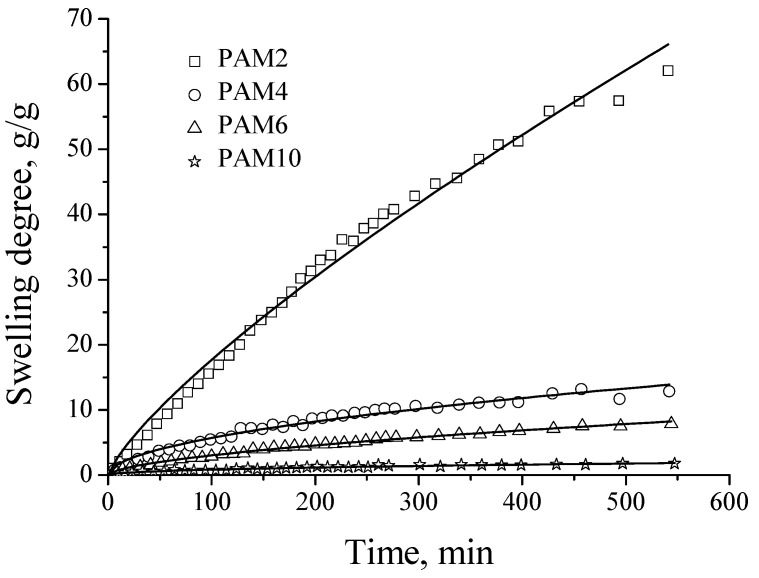
Swelling behavior of the prepared hydrogels (swelling data were fitted to the Korsmeyer–Peppas model).

Experimental data together with the fitted curves according to Equation (2) are shown in Figure 3. The model approximates very well especially the data corresponding to the first 60% solvent uptake in hydrogels. As can be seen in Figure 3 and in Table 2, the model fits the experimental data reasonably well over the whole time interval.

If one compares the SD values obtained after 550 min with the values of SD_eq_ (see Table 1), it is clear that for every single sample the degree of swelling at equilibrium is much higher than that obtained after almost 10 h. This is an obvious consequence of the much higher period of swelling in the first case, but it is also the result of the presence of europium polyoxometalate salt which negatively affects the swelling capacity of the polyelectrolyte in an environment with a substantially increased ionic strength. Moreover, polyoxometalate anions are capable to electrostatically interact with the crosslinker most likely leading to a supplemental physical crosslinking of the polymer network.

The values obtained for *n* indicate that as the concentration of the crosslinking agent increases, the contribution of the relaxation process gradually diminishes and the swelling mechanism tends toward a diffusion one. For the least crosslinked hydrogel PAM2, the relaxation of the polymer network greatly influences the swelling mechanism as M_c_ value is very large and the ability of polymer segments to adopt new conformations during the aqueous solution penetration is tremendous by comparison with the other hydrogels. By contrast, the relaxation process associated with the most crosslinked hydrogel PAM10 is practically insignificant and the swelling follows an almost Fickian mechanism.

### 2.4. Thermal Behavior of Hydogels

**Figure 4:**
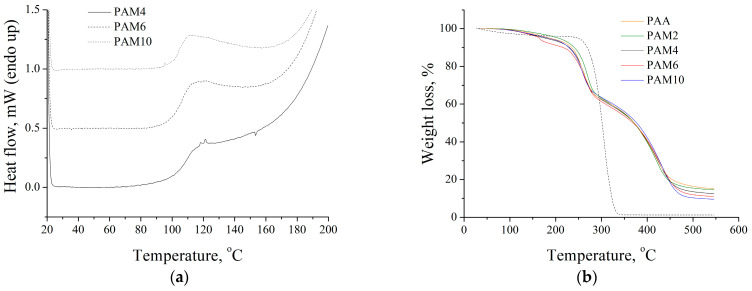
(**a**) DSC curves for polymers PAM2, 6, and 10; (**b**) TG curves for polymers PAA and PAM2-10, together with TG trace for BIBr (dashed line).

Thermal behavior of dry hydrogels was studied by means of differential scanning calorimetry in order to evaluate the changes in flexibility of polymer chains upon the variable content of crosslinking agent. Surprisingly, the glass transition temperature (T_g_) of all copolymers ranged between 105 and 108 °C, almost identical to T_g_ of uncrosslinked poly(acrylic acid), PAA, namely 105 °C. For exemplification, in Figure 4a the DSC thermograms obtained for three xerogels (PAM4, 6, and 10) are plotted and they show minor differences in glass transition temperatures. Our previous study [33] on chemically crosslinked PAA and PAA copolymers obtained via gamma-irradiation showed that both DSC and TG curves were influenced by the absorption dose of radiation, i.e., by the degree of crosslinking. But unlike the gamma irradiation-induced crosslinking, when some additional chemical transformations such as partial decarboxylations accompanied the crosslinkages formation directly between the polymer chains [33], in this case the crosslinking process was mediated by BIBr having an overall flexible structure able to preserve almost the same local mobility of the polymer chains in the newly formed network irrespective of the BIBr content or even when compared to the uncrosslinked system. This means that the corresponding T_g_ values will be very little altered, with tiny differences between them.

At the same time, a similar behavior could be observed when thermal decomposition traces of xerogels were compared to each other and to the uncrosslinked PAA (Figure 4b). As can be seen in Figure 4b, the degradation of all samples followed a three-step process, which can be described as water loss (25–180 °C), reaction between carboxyl groups (200–300 °C), and polymer chain degradation (above 300 °C). The thermal degradation of the crosslinking agent (dashed line in Figure 4b) took place in one important step between 250 and 340 °C (95% weight loss). In more or less the same temperature range interval, decarboxylation, and anhydride formation could contribute to weight loss of all the polymers so it is difficult to observe the individual contribution of BIBr degradation to the total weight loss. Moreover, when the main chain degradation started, BIBr was completely degraded, so again there will be no different degradation profile for PAA when compared to its increasingly BIBr-crosslinked network.

Due to the overlap of the TG thermograms, only the differences in the relative amounts of residue resulted after the degradation completed could be noticed (Figure 4b). Contrarily, in the case of PAA crosslinked via gamma-irradiation, there was no difference between the relative amount of the residue obtained after degradation of uncrosslinked and crosslinked polymers [33]. As the content of BIBr increases in copolymers from PAM2 to PAM10, a less relative amount of residue (from PAA to PAM10) results upon polymers degradation. Indeed, while the residue was 15.11% for PAA, it became 14.60, 12.52, 11.03, and 9.57% for PAM2, PAM4, PAM6, and PAM10, respectively. This behavior is consistent with the increasing percentage of crosslinking agent in the samples. Since almost the whole amount of BIBr vanished before depolymerization started, it is expected to obtain less relative content of residue for the samples containing higher quantities of BIBr.

### 2.5. Rheological Behavior of Poly(Methyl Methacrylate) Latexes

**Figure 5:**
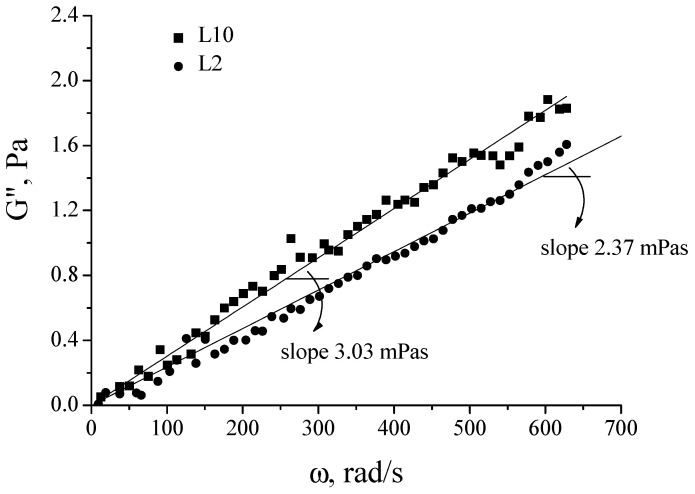
Variation of loss modulus as a function of angular frequency for latexes L2 (●) and L10 (■).

When the same recipes used for preparation of PAM hydrogels were employed for polymerizing an insoluble monomer (methyl methacrylate) via emulsion polymerization, the resulted cationic latexes showed similar macroscopic characteristics no matter the amount of BIBr used. Rheological data revealed a Newtonian behavior for all the prepared latexes, with very similar dynamic viscosities (in the range of 2–3 mPa.s). For better clarity, in Figure 5, the loss modulus (G″) only for latexes L2 and L10 is plotted against angular frequency (ω) and one can notice that for both samples there is a linear dependency of G″ on frequency, which is characteristic to the Newtonian fluids. From the slope of these linear dependencies the dynamic viscosity were calculated, and the obtained values were found to be: 2.37 (for L2), 2.25 (L4), 2.39 (L6), and 3.03 mPa.s (L10).

As the prepared latexes had very low concentrations (around 10%), a Newtonian behavior accompanied by small values of dynamic viscosity was expected to occur. An increase in the amount of surfactant can influence the size and number of particles, and finally the macroscopic viscosity of the latex, but when small concentrations are used, as in this case the differences could be less significant. The highest value of viscosity, obtained for latex L10, having the highest amount of surfactant, can be attributed to a larger number of smaller particles which interact with the aqueous environment through a greater interfacial area, but for all the other samples the differences fell in the range of experimental errors.

**Figure 6:**
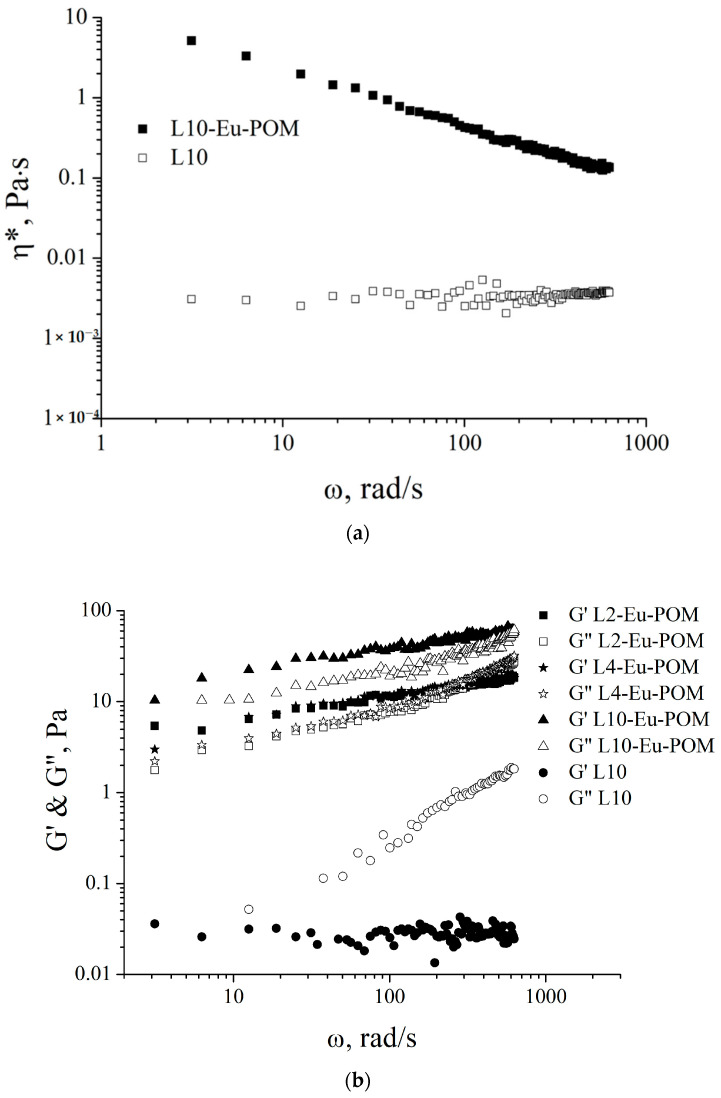
(**a**). Complex viscosity as a function of angular frequency for latex L10 (□) and latex L10-Eu-POM (■); (**b**). G′ and G″ moduli as a function of angular frequency for the indicated samples.

Luminescent latexes obtained after addition of Eu-POM salt displayed much higher viscosities and the rheological behavior changed from Newtonian to pseudoplastic, as can be seen in Figure 6a, where complex viscosity (η*) is plotted against angular frequency, for latex L10 before and after addition of the europium salt. While for latex L10 the complex viscosity is frequency-independent, and has a constant value around 3 mPa.s (the same as dynamic viscosity), for the luminescent latex there is a linear decrease (in a logarithmic scale) of the complex viscosity with increasing the frequency, which corresponds to a pseudoplastic behavior. Additionally, the frequency evolution of G′ and G″ moduli exhibited interesting viscoelastic properties of the investigated latexes with Eu-POM (Figure 6b): the elastic behavior (storage component) prevails with respect to the viscous one (loss component) up to about 200 rad/s (G′ > G″) and then, above this crossover point (ca 200 rad/s), the behavior is reversed (G″ > G′). However, latex L10-Eu-POM is an exception to this general tendency, with a storage component being rather predominant when compared to the energy-dissipative part of the general viscoelastic behavior over the entire range of the angular frequencies applied (G′ > G″). The monotonically increasing course of the loss modulus for the Eu-POM-added latexes, as illustrated in Figure 6b, obeys a low frequency power (G″~ω*^n^*), with values less than unity for the power *n* (*n* = 1 for a Newtonian flow). This is fully in agreement with the pseudoplastic character of the luminescent latexes investigated.

Such a picture could be briefly explained by the relatively weak binder role played by Eu-POM on PMMA particles in the above-mentioned latexes. Indeed, the PMMA latex beads suspended in aqueous environment and carrying positive electric charges (due to their stabilization with BIBr) could be electrostatically bonded through Eu-POM anions over the whole volume of latex resulting a gel-like network having a storage modulus around 50 Pa (for system L10-Eu-POM). The strength of these cohesive forces between PMMA particles via Eu-POM in aqueous phase strongly depends, among other things, on the concentration of the components and their relative abundance in the latexes. That is why the overall effect is not quite large and, irrespective of the BIBr content, the Eu-POM-added latexes behave rather similar: G′ > G″ at ω < 200 rad/s and G″ > G′ at ω > 200 rad/s. Above this crossover point (ω ~ 200 rad/s), the dynamic weak network of latex particles in the presence of Eu-POM species is broken and the energy-dissipative behavior of the system dominates. Nevertheless, this cohesive effect was stronger for latex L10-Eu-POM and, as a result, the dynamic connectivity between the PMMA latex beads and Eu-POM species throughout the latex volume was kept almost unaffected under the oscillatory deformation applied (G′ > G″ for all the angular frequencies used). At the same time, due to the superior cohesiveness electrostatically induced, latex L10-Eu-POM displayed larger G′ and G″ values by comparison with those of the other Eu-POM-added latexes (see Figure 6b). Last but not least, the same higher cohesiveness of Eu-POM-added latexes rationally explains the large difference in the values of dynamic viscosity obtained for Eu-POM-added latexes (high viscous), on one hand, and for Eu-POM-free PMMA latexes (low viscous). 

### 2.6. Luminescence of Eu(III) in Hydrogels and Latexes

The europium-containing polyoxometalates (Eu-POM) show remarkable luminescence performances, such as narrow emission bands, large Stokes shift, quite high luminescence (fluorescence) quantum yield or longer lifetime [34]. Starting from C_4v_ symmetry of [EuW_10_O_36_]^9−^ ions in aqueous solution [35], changes in external microenvironment surrounding Eu^3+^ cations also alter the emission spectra of the complex anions of decatungstoeuropates.

**Figure 7:**
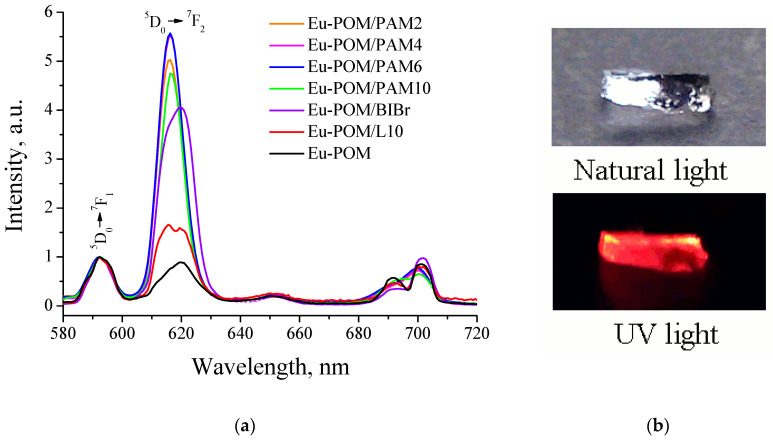
(**a**) Emission spectra for Eu-POM in different environments (λ_ex_ = 365 nm), (**b**) image of PAM10 hydrogel taken in natural and UV light.

Thus, the ^5^D_0_→^7^F_2_ transition (I_2(to F2)_), an electric dipole transition frequently called hypersensitive transition, is extremely sensitive to the small changes in the environment surrounding Eu^3+^ [34,36,37], while the ^5^D_0_→^7^F_1_ transition (I_1(to F1)_) is a magnetic dipole transition whose intensity is quasi-independent of the vicinity of Eu^3+^, being an internal standard of this kinds of Eu^3+^-based compounds [35,36,37]. By taking into account all these features, the value of intensity ratio I_1(to F1)_/I_2(to F2)_ is used as a measure of symmetry/asymmetry of the Eu^3+^ site in different surrounding microenvironments [34,35,36,37,38]. As a general rule, an increase in this ratio value is associated with an increase in Eu^3+^ symmetry and vice versa [36]. For all Eu-POM-containing systems studied, the data analysis was carried out with respect to the pure Eu-POM solution in water for which the ratio value was found to be 1.12. Thus, the emission spectra acquired and normalized to unity for the peak intensities assigned to ^5^D_0_→^7^F_1_ transition (I_1(to F1)_) are collected in Figure 7.

As for our experimental results, beyond the overall decreasing trend of the values of the ratio I_1(to F1)_/I_2(to F2)_ determined for the aqueous mixed systems compared to the pure aqueous solution of Eu-POM, there are two distinct tendencies noticed during the investigation. The first group of examples comprises (a) solutions of gemini surfactant containing Eu-POM and (b) PAM hydrogels soaked in Eu-POM solution. In the case of the mixed aqueous solution of Eu-POM and BIBr, the ratio I_1(to F1)_/I_2(to F2)_ was found to be 0.25, a value well below that characterizing a simple aqueous solution of Eu-POM (1.12). This could be consistent with a partial replacing of water molecules coordinated by the decatungstoeuropate anions with the cationic entities of gemini surfactant in such a way that the higher symmetry of Eu^3+^ surroundings was lowered. A quite similar effect was noticed when the PAM hydrogels were soaked in Eu-POM solution. Thus, I_1(to F1)_/I_2(to F2)_ ranged between 0.18 and 0.21, depending on the relative content of BIBr (mainly as crosslinker). The values of the intensities ratio even lower than those obtained in simple BIBr solution suggest a surfactant coordination to Eu-POM anion sterically hindered as a result of a substantially reduced mobility of BIBr residues covalently linked to the PAM hydrogel structure. On the other hand, the ultrasensitive ^5^D_0_→^7^F_2_ transition of Eu-POM absorbed in PAM hydrogels was detected at 616 nm, practically irrespective of crosslinker content, and for the same species in pure water and in simple BIBr solution—at 619 nm. This corresponds to a clear detectable blue shift effect when Eu-based emission species were transferred into PAM hydrogels. The second group of systems are those of Eu-POM—containing PMMA latexes where, somehow surprisingly, the luminescence spectrum revealed the presence of two peaks located at both 616 and 620 nm. Therefore, the intensity ratios I_1(to F1)_/I_2(to F2)_ were 0.60 and 0.63, respectively which are indicative of a lower symmetry of microenvironment surrounding Eu^3+^ compared to that involved in pure water and a markedly higher symmetry by comparison with that highlighted in the first groups of mixtures. Most likely, the BIBr structures physically and covalently tethered to the PMMA latex beads are, to a relatively great extent, not able to be coordinated to the complex anions [EuW_10_O_36_]^9−^ and, preponderantly, the coordinated water molecules may contribute to the higher symmetry of Eu^3+^ surroundings. However, to give insights into describing this aspect in more details, supplementary investigation requires to be pursued.

**Figure 8:**
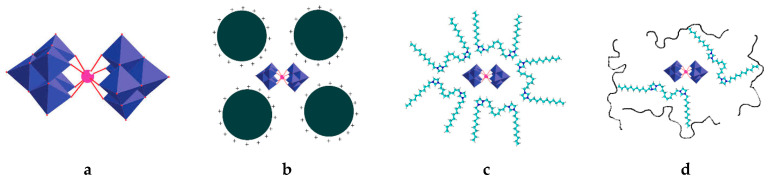
Schematic representation of different environment for Eu-POM: (**a**) water; (**b**) PMMA latex; (**c**) aqueous BIBr solution; (**d**) PAM hydrogel.

In Figure 8 the structure of Eu-POM anion and the proposed arrangements in different surroundings are schematically depicted.

## 3. Conclusions

The prepared dicationic gemini surfactant (BIBr) having a relatively large linear polymethylene chain spacer (10 methylene groups) and two double bonds at the end of each of the two side chains grafted on the imidazolium rings was proved to be capable of forming premicellar aggregates sensed by pyrene probe at concentrations three times lower its real critical micelle concentration. Thermodynamic parameters indicated a pronounce spontaneous character of micellization, and despite the long connecting chain between the ionic groups the degree of ionization in the micelles was 0.58.

When Eu-POM anions were dissolved in the micellar solution of BIBr, attractive electrostatic interactions between the polyanions and the cationic groups of surfactant created a surfactant-rich layer in the close vicinity of every single Eu-POM anion (by a partial replacement of water from the coordination microenvironment), with a direct consequence in drastically decreased symmetry of Eu^3+^ site by comparison with that found in pure water. This result is based on the values of the intensity ratio I_1(to F1)_/I_2(to F2)_ experimentally found for the luminescence emission of the Eu-based complex anions in different environments: 1.12 in pure water and 0.25 in aqueous solution of BIBr.

Due to its two terminal double bonds, the gemini surfactant was used as crosslinker to prepare acrylic acid-based hydrogels, via solution polymerization. For the hydrogel having the highest degree of crosslinking, the swelling mechanism was controlled by diffusion, whilst for hydrogels having more loosen networks, the relaxation of polymer network played the most important role in the swelling process. Thermal behavior of the dry hydrogels indicates that despite the different crosslinking density of the hydrogels the glass transition temperature slightly changed and also no significant alteration in their thermal stability was observed.

The dicationic surfactant was also used to obtain latex particles charged with cationic groups, chemically and physically bound to the surface of the poly(methyl methacrylate) beads. Adding Eu-POM salt to PMMA latexes resulted in an increase in the viscosity of the system, as well as in changing the rheological behavior from a Newtonian to a pseudoplastic one due to the attractive electrostatic interactions between the polyoxometalate anions and the positively charged polymer particles.

Electrostatic bonding of Eu-POM to polymeric matrices, such as poly(acrylic acid) hydrogels or poly(methyl methacrylate) latexes, modifies the ratio between the intensities of ^5^D_0_→^7^F_1_ and ^5^D_0_→^7^F_2_ transitions in Eu^3+^ emission spectrum. This proves that there is a change in the symmetry of the microenvironment around the lanthanide ion in the sense of its decreasing by comparison with what was observed in water. A number of interesting findings strongly related to the interactions between cationic gemini surfactant and anionic structure of Eu-POM, such as the presence of two distinct emission peaks assigned to the hypersensitive ^5^D_0_→^7^F_2_ transition located at about 616 and 620 nm, the blue shift effect shown by the wavelength of the same sensitive transition when emissive Eu-POM was transferred from its aqueous solution or in a mixed solution with BIBr to a polymer environment, and other adjacent peculiarities, are required to be further investigated.

## 4. Materials and Methods

### 4.1. Materials

11-bromo-1-undecene (95%, Sigma-Aldrich, Switzerland) and commercial grade solvents were used as received. 1,1′-(1,10-decanediyl) bisimidazole was prepared following literature procedures [39], the one used here being described in our previous papers [40,41,42,43].

Eu-polyoxometalate Na_9_EuW_10_O_36_ was freshly prepared according to a procedure reported elsewhere [44]. Briefly Na_2_WO_4_^.^2H_2_O was dissolved in water and the pH of the solution was adjusted to 7–7.5 using acetic acid. An aqueous solution of Eu(NO_3_)_3_^.^6H_2_O was added drop wise to the above-mentioned solution under stirring at 80–90 °C. After cooling, filtering, and drying, colorless crystals of Na_9_[EuW_10_O_36_]^.^32H_2_O were obtained.

Acrylic acid (≥99%, Fluka, Buchs, Germany), AA, and methyl methacrylate (≥99%, Fluka, Buchs, Germany), MMA, as monomers, and 2,2′-azobis(2-methylpropionamidine) dihydrochloride (97%, Sigma-Aldrich, Germany), V50, as initiator were used as received.

### 4.2. Synthesis and Characterization of 1,1′-(1,10-Decanediyl)-Bis [3-(Undec-10-en-1-yl)-Imidazolium] Bromide Salt, BIBr

All the chemicals were used as supplied. 1,1′-(1,10-decanediyl) bisimidazole (3.2 g, 11.7 mmol) was dissolved in acetonitrile (25 mL). To this solution, 11-bromo-1-undecene (6.1 g, 26.2 mmol) was added, and the reaction mixture was heated under reflux for 24 h. After this time, the solution was concentrated via rotary evaporation to about half of its initial volume and ethyl ether (30 mL) was added to obtain a white solid. The crude product was recrystallized from a mixture of dichloromethane/ethyl ether (1/2, *v/v*) and further dried in vacuum. Light yellow waxy solid was obtained (yield 60%).

The chemical structure of the bis-imidazolium salt, depicted in Scheme 1, was in agreement with the data obtained by ^1^H and ^13^C NMR, elemental analysis, and IR spectroscopy (see Figures S1–S3). CHN elemental analysis was carried out with a EuroEA 3300 instrument. IR spectra were recorded on a Bruker Tensor V-37 spectrophotometer using KBr pellets in the range of 4000–400 cm^−1^. ^1^H and ^13^C NMR spectra were acquired by employing a Bruker Fourier 300 spectrometer operated at 500 MHz, using CDCl_3_ as solvent. ^1^H and ^13^C chemical shifts were referenced to the solvent peak position: δ 7.26 ppm ^1^H, and 77.00 ppm ^13^C.

^1^H-NMR (300 MHz, CDCl_3_, ppm): 10.34 (s, 2H), 7.69 (s, 2H), 7.43 (s, 2H), 5.75 (m, 2H), 4.97 (m, 1H), 4.90 (m, 2H), 4.86 (m, 1H), 4.32 (m, 8H), 1.94 (m, 12H), 1.26 (m, 42H).

^13^C-NMR (75 MHz, CDCl_3_, ppm): 139.02, 136.71, 122.57, 121.84, 114.07, 49.93, 49.75, 33.64, 30.24, 29.85, 29.20, 28.91, 28.87, 28.74, 28.38, 28.05, 26.13, 25.55.

IR (ATR, cm^−1^): 3450, 3127, 2910, 2851, 2154, 2009, 1639, 1555, 1467, 1152, 854, 752, 556.

Two methods were used for critical micelle concentration (cmc) evaluation: conductometric titration and I_1_/I_3_ rule of pyrene as fluorescence probe. Stock solutions with concentration 11.14 mM BIBr were prepared in distilled water.

The conductometric titrations were performed by measuring specific conductivities (pH/conductometer Consort C861) over a corresponding range of surfactant concentrations using the initial stock solution of BIBr as titrant. The value for cmc was considered as the abscissa of the intersection point between the two tangents to the linear dependencies of the specific conductivity—surfactant concentration plot before and after micellization.

As for determining cmc via fluorescent probes method, an aqueous solution of about 2 × 10^−6^ M of pyrene was prepared from a stock solution of pyrene in ethanol following an algorithm described elsewhere [45]. The resulted aqueous solution of pyrene was utilized as solvent for samples having different concentrations of BIBr. All fluorescence measurements were recorded on a Jasco FP-6300 spectrofluorimeter. The cmc was accounted as the abscissa of the inflection point of the sigmoidal dependence of I_1_/I_3_ ratio on surfactant concentration.

### 4.3. Synthesis and Characterization of PAM Hydrogels Using BIBr as Crosslinking Agent

Copolymerization of AA and BIBr was performed in distilled water by radical polymerization using 2,2′-azobis(2-methylpropionamidine) dihydrochloride (V50) as an initiator (2 wt.% with respect to the amount of monomers). The amount of monomer (AA) was 10 wt.%, while BIBr was added in different ratios for crosslinking the polymer chains. All ingredients were added in sealed vials and introduced in water bath under vigorous stirring and constant temperature of 70 °C to initiate and evolve the polymerization process for 4 h.

After polymerization, the obtained hydrogels were washed in distilled water for several days in order to remove all soluble products: unreacted monomers, initiator, or soluble oligomer fractions. Finally, the samples were left to swell until equilibrium was reached. From time to time, swollen hydrogels were withdrawn from water, wiped with filter paper for removing the excess of surface water, and weighted. The procedure was repeated until a constant mass is obtained (one week). Then, the hydrogels were dried in oven at 50 °C to constant mass. The xerogels were also weighted and the equilibrium swelling degree was calculated as follows:

(3) $$SD_{eq}=\frac{m_{s}-m_{d}}{m_{d}}$$

where m_s_ is the weight of the hydrogel at equilibrium swelling and m_d_ is the weight of xerogel. The swelling procedure was performed for three samples from each xerogel and the mean values were considered. In all cases, the values of the relative standard deviation were less than 15%.

Xerogels density was determined using a Mettler Toledo AG204 balance equipped with density determination kit. The samples were weighted in air and in distilled water at room temperature and the polymer density, ρ_p_, was calculated using the following equation:

(4) $$\rho_{p}=\frac{m_{air}\rho_{w}}{m_{air}-m_{w}}$$

where m_air,w_ is the mass of xerogel weighted in air, and in water, respectively, while ρ_w_ is the density of water at room temperature. The final values were the average of three measurements. Thus, the density of PAM2 was found to be 1.26 ± 0.05 g/cm^3^, which is close to 1.2 g/cm^3^, the density of poly(acrylic acid). The other xerogels displayed practically identical densities of 1.35 ± 0.02 g/cm^3^.

The average molecular mass between two consecutive crosslinking points was calculated using Flory–Rehner equation [46]:

(5) $${\bar{M}}_{c}=-\frac{\rho_{p}V_{1}\left(\varphi_{2}^{1/3}-\varphi_{2}/2\right)}{\ln(1-\varphi_{2})+\varphi_{2}+\chi \varphi_{2}^{2}}$$

with V_1_—the molar volume of water, φ_2_—the volume fraction of polymer, and χ—the polymer–solvent interaction parameter. As water is a good solvent for poly(acrylic acid), the interaction parameter was evaluated as [47]:

(6) $$\chi =\frac{\ln(1-\varphi_{2})+\varphi_{2}}{\varphi_{2}^{2}}$$

with φ_2_ is given by:

(7) $$\varphi_{2}=\frac{1}{1+\frac{\rho_{x}}{\rho_{w}}SD_{eq}}$$

where ρ_x_ is the density of xerogel. The mean value obtained for SD_eq_ was used in Equation (7).

The influence of the degree of crosslinking on thermal behavior of the polymers was investigated using a Thermogravimetric Analyzer Q50 (TA Instruments) using alumina crucibles, under nitrogen atmosphere, from room temperature to 550 °C with a heating rate of 10 K/min.

Differential scanning calorimetry (DSC) investigation was performed for all xerogels on a Diamond DSC Perkin-Elmer instrument under nitrogen purging at a heating rate of 10 K/min between 20 and 180 °C. Glass transition temperature (T_g_) was considered the abscissa of the inflection point on the heat flow vs. temperature curve, obtained during the second cycle of heating.

### 4.4. Emulsion Polymerization of PMMA Using BIBr as Surfmer

The same recipes were used for emulsion polymerization of MMA, and the resulted latexes were denoted as L2, L4, L6, and L10, in accordance with the percentage of BIBr used for their preparation (wt.% with respect to monomer amount). First of all, V50 and BIBr were dissolved in distilled water. As MMA is almost insoluble in water, a tiny amount of monomer was dissolved in water, while its majority was dispersed as droplets stabilized by surfactant (BIBr) adsorption onto their surface. Moreover, a very small amount of MMA was solubilized in the nonpolar core of BIBr micelles, where most of the initiation and polymerization reactions took place. The synthesis was performed following the same procedure as for solution polymerization. The resulted latexes were cleaned by dialysis against distilled water (until the water conductivity reached a minimum value and no longer varied) in order to remove unreacted initiator, surfactant, or even monomer.

### 4.5. Luminescence of Europium (III) Probes in Different Environments

Luminescent hydrogels were prepared by swelling the PAM xerogels with aqueous solutions of Na_9_EuW_10_O_36_^.^32H_2_O (Eu-POM). Swelling kinetics was investigated for all the prepared gels for a period of almost 10 h. Samples of xerogels were weighted, then placed in aqueous solutions of Eu-POM, and let to swell at room temperature. At specific time intervals, the samples were removed from solution, wiped with filter paper, and weighted. The degree of swelling was calculated and plotted against time.

For the PMMA latexes, calculated amounts of Eu-POM were added to each latex to ensure a stoechiometric ratio between bisimidazolium salt and europium salt. The changes in viscosity and rheological behavior of the latexes after addition of Eu-POM were revealed by dynamic rheology performed on a Micro Fourier Rheometer MFR 2100 (GBC, Australia). The rheological measurements were carried out at room temperature, the samples being placed between the two parallel plates of the rheometer, which is capable to apply a random displacement (by means of the upper plate) with a broadband frequency. The amplitude of the deformation vertically applied was kept low enough as to ensure a linear viscolastic response of the samples. Both storage (G′) and loss (G″) moduli were obtained as a function of frequency of squeezing deformation used. The operational parameters for all the investigated samples were: gap between the parallel plates: 300 μm, frequency range: 0.5–100 Hz, displacement amplitude: 0.03 μm.

The photoluminescence spectra were recorded on an Ocean Optics QE65PRO spectrometer connected via optical fiber with a polarizing optical microscope (Nikon 50 iPol) by collecting the light emission resulted after the excitation of samples through the microscope objective. A Nikon Intensilight and an LLS-LED (Ocean Optics, 365 nm) integrated with the microscope were used as excitation sources.

## Figures and Tables

**Table 1:** Hydrogels characteristics.

	PAM2	PAM4	PAM6	PAM10
SD_eq_ (g/g)	1070	306	58.2	18.6
φ_2_^.^10^3^	0.70	2.42	12.50	38.00
M_c_^.^10^−5^ (g/mol)	37.10	5.52	0.35	0.05

**Table 2:** Swelling parameters (Korsmeyer–Peppas model) of the prepared hydrogels.

Hydrogel	k, min^−*n*^	*n*	R^2^
PAM2	0.49 ± 0.05	0.78 ± 0.02	0.99078
PAM4	0.51 ± 0.05	0.53 ± 0.02	0.97795
PAM6	0.12 ± 0.01	0.59 ± 0.01	0.99363
PAM10	0.10 ± 0.01	0.47 ± 0.02	0.95342

## Data Availability

Not applicable.

## Supplementary Materials

The following supporting information can be downloaded at: https://www.mdpi.com/article/10.3390/gels8120786/s1.
