# Supplementary material for: Novel Gemini Surfactant for Binding Eu(III)-Polyoxometalate into Hydrogels and Polymer Latexes
Source: Gels. 2022 Nov 30;8(12):786. doi: 10.3390/gels8120786 (PMC9777751; doi:10.3390/gels8120786)
Supplement: Supplementary file 1 [file gels-08-00786-s001.zip › gels-2060015-supplementary Materials.pdf]

gels

Supplementary Materials

Novel Gemini Surfactant for Binding Eu(III)-Polyoxometalate into Hydrogels and Polymer Latexes

Marin Micutz [1], Viorel Circu [2], Monica Ilis [2] and Teodora Staicu [1,*]

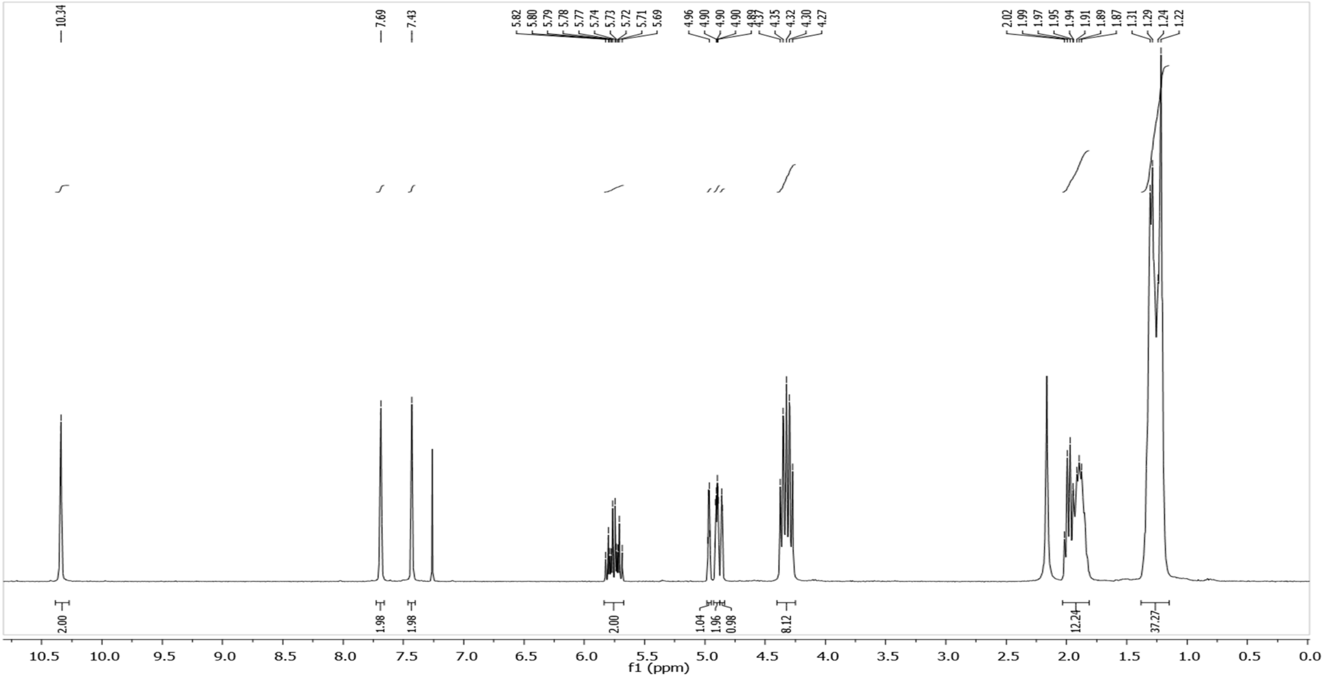

Figure S1. ^{1}H NMR spectrum for 1,1`-(1,10-decanediyl)-bis[3-(undec-10-en-1-yl)-imidazolium] bromide salt, BIBr.

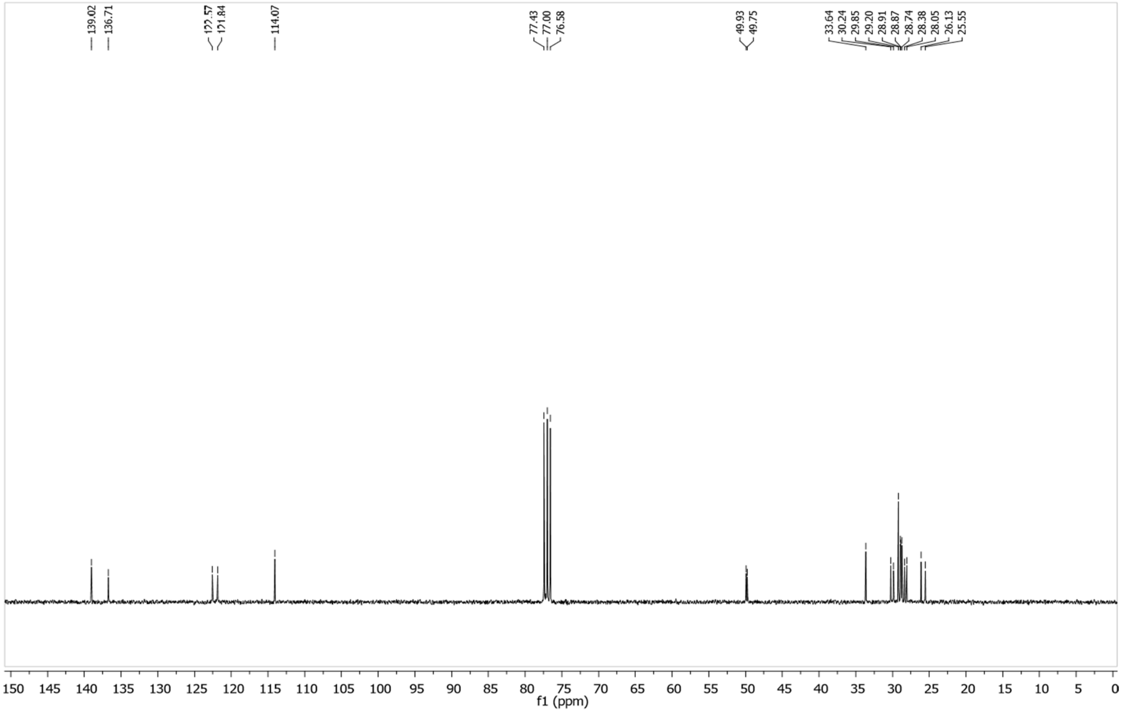

Figure S2. ^{13}C NMR spectrum for BIBr.

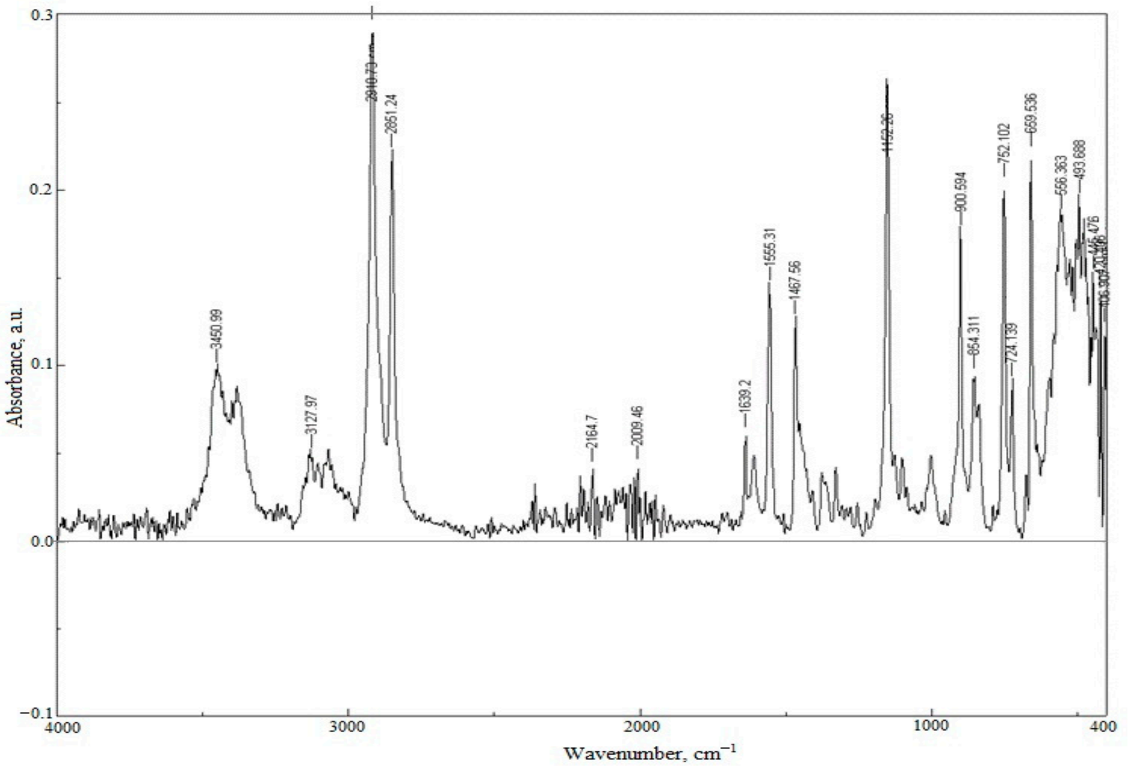

Figure S3. IR spectrum for BIBr.
